# CRISPR-Cas9-Based Technology for Studying Enteric Virus Infection

**DOI:** 10.3389/fgeed.2022.888878

**Published:** 2022-06-08

**Authors:** Junki Hirano, Kosuke Murakami, Tsuyoshi Hayashi

**Affiliations:** Department of Virology II, National Institute of Infectious Diseases, Tokyo, Japan

**Keywords:** CRISPR-Cas9, genome wide screen, host factors, enteric virus, norovirus, rotavirus, gene knockout

## Abstract

Enteric viruses, including numerous viruses that initiate infection in enteric canal, are recognized as important agents that cause wide spectrum of illnesses in humans, depending on the virus type. They are mainly transmitted by fecal-oral route with several vector such as contaminated water or food. Infections by enteric viruses, such as noroviruses and rotaviruses, frequently cause widespread acute gastroenteritis, leading to significant health and economic burdens and therefore remain a public health concern. Like other viruses, enteric viruses ‘‘hijack’’ certain host factors (so called pro-viral factors) for replication in infected cells, while escaping the host defense system by antagonizing host anti-viral factors. Identification(s) of these factors is needed to better understand the molecular mechanisms underlying viral replication and pathogenicity, which will aid the development of efficient antiviral strategies. Recently, the advancement of genome-editing technology, especially the clustered regularly interspaced short palindromic repeat (CRISPR)-Cas9 system, has precipitated numerous breakthroughs across the field of virology, including enteric virus research. For instance, unbiased genome-wide screening employing the CRISPR-Cas9 system has successfully identified a number of previously unrecognized host factors associated with infection by clinically relevant enteric viruses. In this review, we briefly introduce the common techniques of the CRISPR-Cas9 system applied to virological studies and discuss the major findings using this system for studying enteric virus infection.

## Introduction

Acute gastroenteritis (AGE) is a disease with symptoms including vomiting, watery diarrhea, stomach pain and dehydration, and is frequently caused by intestinal infection with pathogenic bacteria, viruses, or parasites (Graves, 2013; Bányai et al., 2018; Santos-Ferreira et al., 2021). Among these agents, enteric viruses, particularly caliciviruses (e.g., norovirus and sapovirus), rotavirus, astrovirus, and enteric adenovirus are responsible for viral AGE (Bishop and Kirkwood, 2008; Makimaa et al., 2020). In addition, enteroviruses (EVs), which generally express their virulence in other organs, are often detected in diarrheal feces and are thought to be positively linked to AGE. Viral gastroenteritis in healthy individuals is normally self-resolving, whereas vulnerable populations, including infants, elderly, and immunodeficient or immunocompromised individuals are at risk of chronic gastroenteritis and severe complications accompanied by prolonged viral shedding (Santos-Ferreira et al., 2021). Despite their clinical relevance, only a few vaccines (e.g., rotavirus vaccines) are available. Additionally, no antiviral drugs to prevent or treat viral gastroenteritis have been developed to date (Santos-Ferreira et al., 2021).

Similar to other viruses, enteric viruses rely on their hosts to successfully multiply in infected cells. Characterization of viral-host interactions is necessary to gain a clear view of the infection and replication mechanisms, which will contribute to developing host-directed drugs against pathogenic viruses. Genome-wide genetic screening is one of the most powerful and rapid tools to uncover new insights into the viral lifecycle through a comprehensive search for host factors associated with viral infection (Puschnik et al., 2017; Lin et al., 2021). Recently, a novel technology, called clustered regularly interspaced short palindromic repeat (CRISPR)-Cas9 system made a breakthrough in screening approach. In this review, we introduce the basics and common techniques of the CRISPR-Cas9 system and then describe how this system contributes to expanding the knowledge of enteric virus infection.

## Overview of CRISPR-Cas9 System

**FIGURE 1:**
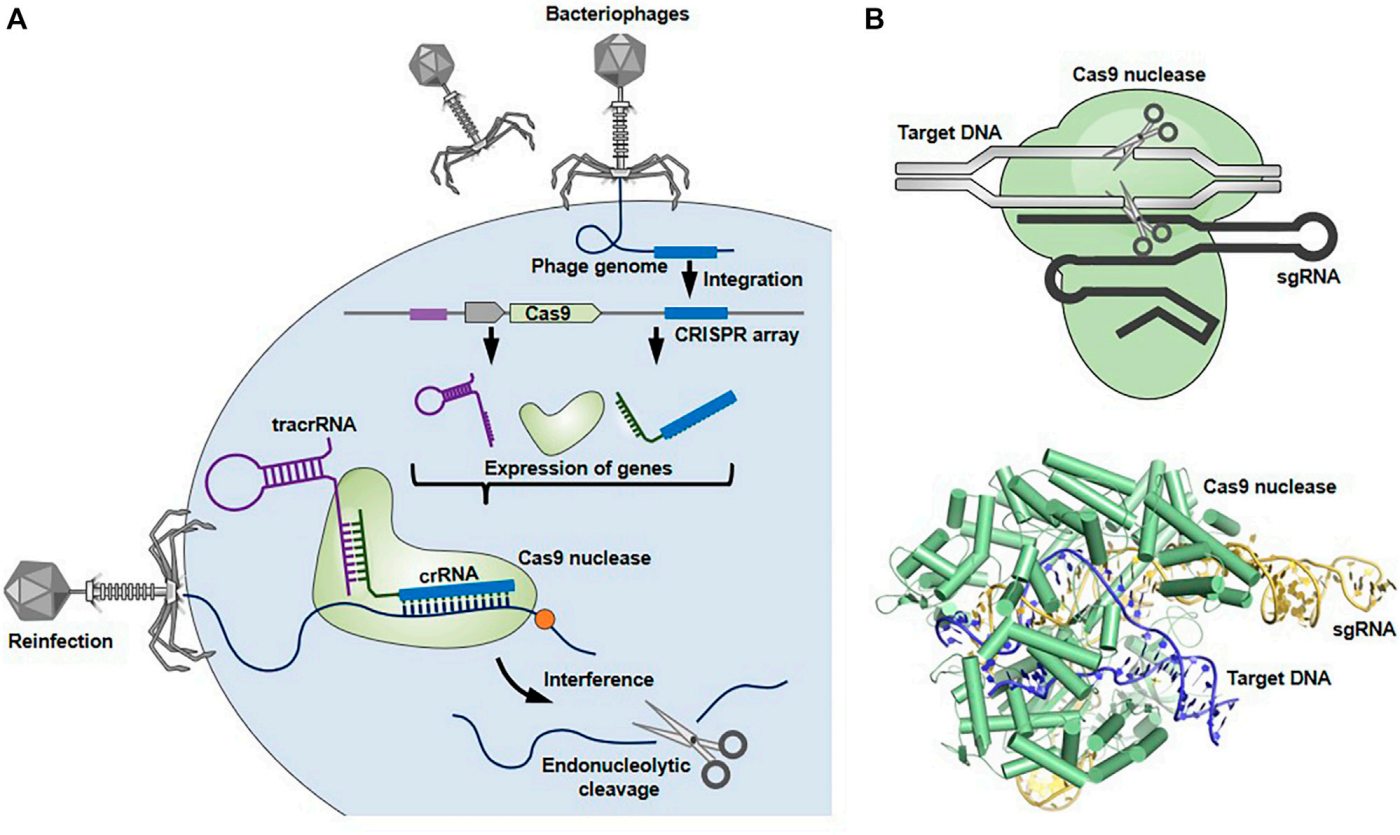
Bacterial CRISPR-Cas9 system. **(A)** CRISPR-Cas9 system works as a host defense system. When an invader (e.g., bacteriophage) infects bacterial cells, spacer-sized DNAs derived from the invader’s genome are incorporated into the CRISPR locus of the bacterial genome. Upon subsequent infection, the gRNA-Cas9 complex recognizes and cleaves the target genome sequences of re-invading pathogens to protect the bacteria from lethal infection. **(B)** The structures of gRNA-Cas9 complex (Protein Data Bank in Europe, 5F9R) were drawn using the open-source PyMOL Molecular Graphics System version 1.8.6.0.

The CRISPR-Cas9 system was originally discovered as an adaptive immune system utilized by bacteria and archaea against infection of bacteriophages and plasmids (Figure 1) (Ishino et al., 2018; Ghorbani et al., 2021). This system consists of three steps: 1) acquisition, 2) expression, and 3) interference, where a Cas9 nuclease and two short RNAs, called CRISPR-RNA (crRNA) and transactivating RNA (tracrRNA), coordinately function as scissors and guide, respectively. 1) In the acquisition step, genomic DNA fragments derived from invading bacteriophages or plasmids are incorporated into the CRISPR locus of a bacterial genome as a new ‘‘spacer’’ separated by repeated sequences. This spacer sequences, conserving a part of the invader’s genome, act as an immunological memory thereafter. 2) The crRNA is transcribed from a corresponding spacer at the CRISPR locus. The tracrRNA and Cas9 nuclease, which are other key players in this host defense machinery, are also expressed from the CRISPR locus. The crRNA and tracrRNA form a hybrid, so-called guide RNA (gRNA), by binding the complementary repeated sequences, forming a complex with Cas9. 3) The gRNA-Cas9 complex targets complementary sequences of the re-invading pathogen’s genomic DNA, leading to the creation of a double stranded break (DSB) in the RNA-guided targeted genome element, resulting in its elimination. Cas9-mediated cleavage occurs only when a protospacer adjacent motif (PAM) sequence (e.g., NGG for *Streptococcus pyogenes* Cas9) is located upstream of the target nucleotides, which grants specific cleavage of non-self DNA (Puschnik et al., 2017; Ishino et al., 2018; Lin et al., 2021).

In the past decade, this CRISPR-Cas9 system has been successfully adopted in mammalian cells and is currently pervaded as a common technique for generating gene knockout mammalian cells (Cong et al., 2013; Jinek et al., 2013; Mali et al., 2013). Cas9 has been human codon-optimized and fused to a nuclear localization signal to direct it to the nucleus. Expression of the modified Cas9 together with gRNA, consisting of synthesized crRNA and tracrRNA sequences that target certain gene(s), allows the creation of a DSB in RNA-guided gene sequences in mammalian cells. The DSB is subsequently repaired by the nonhomologous end joining (NHEJ) pathway, which often makes frameshift mutations in the targeted gene, thereby leading to translation of truncated or non-functional proteins, resulting in gene knockout.

The CRISPR-Cas9 system has been diverted to gene overexpression as a CRISPR-catalytically dead Cas (dCas) activation system (Brezgin et al., 2019; Chong et al., 2020). dCas is able to bind target nucleotides guided by the gRNA, while unable to create DSB into the DNA due to the mutations in its nuclease domain (Brezgin et al., 2019; Chong et al., 2020). In this system, dCas is fused to transcriptional activators such as herpes simplex virus VP16 activation domain (VP64). The dCas9-VP64-gRNA complex activates RNA guided-promoter sequences and thereby upregulates expression of endogenous genes of interest in mammalian cells, in contrast to ectopic overexpression by foreign cDNA introduction.

## CRISPR-Cas9 as a Genome-wide Genetic Screening Tool for Studying Virology

Unbiased genome-wide genetic screening is one of the gold standard methods to comprehensively identify host factors that restrict or promote viral infection (Puschnik et al., 2017; Chulanov et al., 2021). There are two types of forward genetic screens, loss-of-function and gain-of-function screens. The loss-of-function screens, which have been conducted so far, are based on transient (siRNA) or stable (shRNA) knockdown of gene expression, or complete gene knockout, but only applicable in haploid and near-haploid cells. In contrast, a loss-of-function screen employing CRISPR-Cas9 enables complete gene knockout in a wide variety of cells, thus providing a strong advantage compared to RNAi (siRNA or shRNA) or haploid-based screens (Puschnik et al., 2017; Chulanov et al., 2021). Gain-of-function screening relies on ectopic overexpression by the introduction of a cDNA library. The sgRNA library combined with dCas can be used for a gain-of-function screen, called a CRISPR activation screen, requiring less effort than a traditional cDNA library which needs to clone a vast number of individual human genes into a vector beforehand (Puschnik et al., 2017; Chulanov et al., 2021).

**FIGURE 2:**
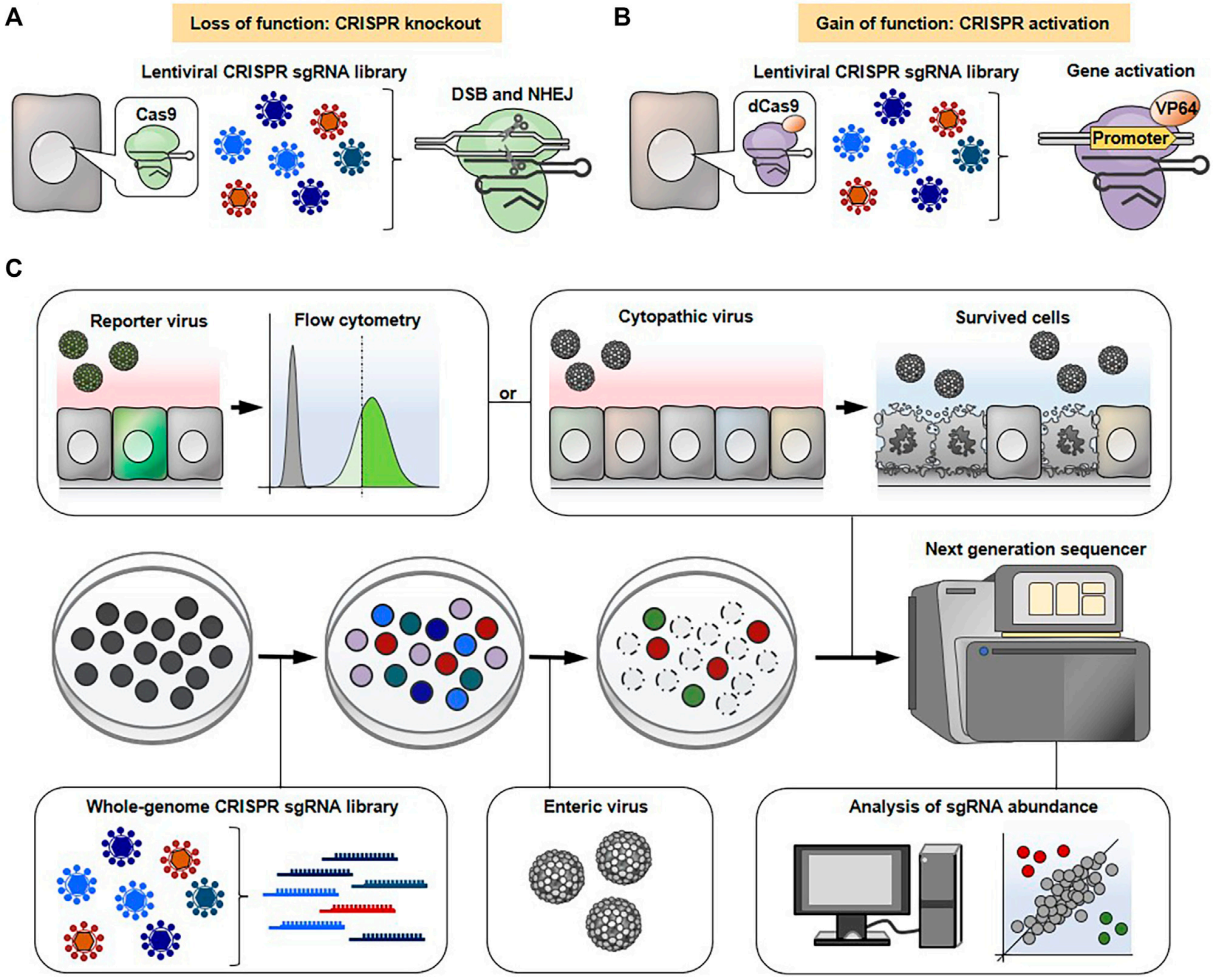
General workflow for genome-wide CRISPR-Cas9 screens. CRISPR-Cas9 **(A)** or CRISPR-dCas system **(B)** is used to conduct loss-of-function **(A)** or gain-of-function **(B)** screens, respectively. **(C)** The cells are transduced with lentivirus containing either a CRISPR knockout sgRNA library **(A)** or an activation sgRNA library **(B)**, followed by a cytopathic virus infection. The surviving cells that are expected to confer resistance to lethal infection due to specific gene knockout or overexpression are collected. The sgRNA abundance in surviving cells and uninfected control cells is determined by next generation sequencing to narrow down the gene(s) associated with phenotypic changes. Alternatively, the cells are infected with a reporter virus (e.g., GFP or mCherry) and the cells expressing the viral gene at high or low levels are sorted using flow cytometry, followed by next generation sequencing.

The typical experimental flow of a genome-wide CRISPR-Cas9 screen is shown in Figure 2. The cells are transduced with lentivirus carrying either a genome-wide CRISPR knockout or an activation library to knockout or activate each gene on a genome-scale, respectively. Various types of pre-made CRISPR sgRNA libraries aimed at gene knockout or activation are commercially available. The pooled mutagenized cell populations are then infected with a cytopathic virus. Subsequently, the surviving cells, which are expected to confer resistance to viral infection due to knockout or overexpression of certain gene(s) are harvested. The enrichment of sgRNA sequence(s) in surviving cells and uninfected control cells are comprehensively compared using next generation sequencing followed by bioinformatics analysis to find gene(s) associated with phenotypic changes. It should be noted that not all viruses are able to induce cytotoxicity in susceptible cells. In such cases, flow cytometry-based selection can be applicable for screening. The cells are infected with a reporter virus, such as a green fluorescent protein (GFP)-expressing virus, and the cells expressing the viral gene at high or low levels are enriched by flow cytometry followed by next generation sequencing. Alternatively, the infected cells can be stained with fluorescently-labeled viral antibody and then subjected to flow cytometry-based selection.

The cell type used for screening can be selected depending on the experimental design and/or available resources (Puschnik et al., 2017). For instance, a knockout screen using cells susceptible to a cytopathic virus, an infection condition where almost all cells (>99%) die after viral infection, followed by an analysis of the surviving cells is biased in favor of identifying pro-viral factor(s), including entry receptor(s). Using the same sets of virus and cells, but a condition where approximately half of the cells are dead with viral infection, potentially targets both pro- and anti-viral factors. An activation screen using non-susceptible cells followed by analysis of virus-positive cells would most likely lead to identifying pro-viral factor(s). Generally speaking, pro-viral or anti-viral host factors, which promote or restrict any step of the viral life cycle (e.g., virus entry, RNA replication, protein translation, assembly, or virus egress), can be identified in a CRISPR-Cas9 screen. The potentially identified pro-viral factor(s) that target viral entry include a cellular receptor for a virus (e.g., CD4 for human immunodeficiency virus) or its stimulator, while expected anti-viral factor(s) targeting virus entry include host protein(s) that bind to virions for inhibiting its entry into the cells or downregulate expression of virus entry receptor.

In the following sections, we summarize important findings pertaining to each enteric virus infection, mainly focusing on insights obtained from genome-wide CRISPR-Cas9 genetic screens.

### Norovirus

Norovirus belongs to the genus *Norovirus* (family Caliciviridae) and contains a positive-sense, single-stranded RNA genome (Robilotti et al., 2015; de Graaf et al., 2016). Human norovirus (HuNoV) is a highly contagious pathogen and a major cause of acute gastroenteritis and foodborne diseases worldwide, affecting all age groups (Bányai et al., 2018). Although gastroenteritis caused by HuNoV infection is self-limiting, with durations of several days to a week in healthy individuals, people with compromised or weakened immune systems potentially develop chronic gastroenteritis due to persistent HuNoV infection that can last weeks, months, or even years (Green, 2014; Santos-Ferreira et al., 2021).

Despite its clinical importance, the understanding of HuNoV biology has been hampered by the lack of an *in vitro* HuNoV cultivation system until recently. Therefore, murine norovirus (MNV), which belongs to the same genus as HuNoV, has been used as a surrogate virus to understand the molecular mechanism underlying HuNoV infection. MNV can grow robustly in the RAW264.7 murine macrophage cell line with a cytopathic effect. Using this MNV-culture system, unbiased genome-wide CRISPR-Cas9 knockout screens successfully identified CD300lf and CD300ld as functional entry receptors for MNV (Haga et al., 2016; Orchard et al., 2016). It has been later reported that a human CD300 ortholog (CD300lf) does not function as HuNoV’s entry receptor, indicating that HuNoV and MNV appear to use different mechanisms for host cell entry (Graziano et al., 2020).

Aside from the identification of an entry receptor, CRISPR-Cas9 knockout screens have also contributed to identifying a novel pro-MNV host factor; stress granule component G3BP1, which enhances viral VPg-dependent translation (Hosmillo et al., 2019). Furthermore, Orchard *et al.* conducted a genome-wide CRISPR activation screening using HeLa cells expressing CD300lf and found 49 genes that restrict MNV replication in human cells when overexpressed (Orchard et al., 2019). These include known anti-viral genes (e.g., Mx1 and IFITM1), as well as previously unrecognized genes related to anti-viral activity. One such gene, TRIM7 was found to inhibit MNV replication by targeting a post-entry step. It remains unclear whether these host factors also play a role in HuNoV infection.

Recently, several successive HuNoV cultivation systems have been developed (Jones et al., 2014; Ettayebi et al., 2016; Sato et al., 2019; Van Dycke et al., 2019), one of which is a culture system employing tissue stem cell-derived human intestinal enteroids (HIEs) (Ettayebi et al., 2016; Ettayebi et al., 2021). Given the above-mentioned advances with respect to MNV biology, there is no doubt that the CRISPR-Cas9 system could also become a good weapon for studying HuNoV infection. Indeed, using this system, Lin et al., very recently developed knockout HIE lines for genes related to innate immunity (e.g., STAT1 or the interferon receptor) by means of the CRISPR-Cas9 system and found that interferon pathways control GII.3 human norovirus infection in HIEs (Lin et al., 2020; Lin et al., 2022). The HuNoV research using a *de novo* infection system is still in its infancy, and it is safe to assume that numerous host factors related to HuNoV infection remain to be identified. It would be worth investigating whether the previously identified pro- and anti-MNV factors, including those mentioned above, are involved in HuNoV infections. Further candidate approaches (e.g., pro- or anti-MNV factors) and unbiased genome wide screens utilizing the CRISPR-Cas9 system should be highly encouraged to gain further insights regarding the HuNoV infection mechanism.

### Rotavirus

Rotavirus belongs to the genus *Rotavirus* (family Reoviridae) and is a non-enveloped RNA virus whose genome consists of 11 segmented double-stranded RNAs. Human rotavirus (HRV) primarily infects children under 5 years of age and often induces severe and dehydrating gastroenteritis (Crawford et al., 2017; Bányai et al., 2018). Rotavirus vaccines (RotaTeq^®^ and Rotarix^®^) were introduced globally over a decade ago, exhibiting high efficacy in preventing rotavirus-related diseases. However, HRV-associated gastroenteritis still leads to >200,000 deaths annually, mostly in low-income countries, possibly because of less availability of rotavirus vaccines and dehydration therapy and lower efficacy of vaccines than those in high-income countries (Bányai et al., 2018; Santos-Ferreira et al., 2021).

*In vitro* studies of viral-host interactions have been conducted mainly using animal rotaviruses (e.g., simian rotavirus), which grow robustly in transformed mammalian cell lines, including monkey cell lines (e.g., MA104 and Vero) and human intestinal epithelial cell lines (e.g., Caco-2 and HT-29). Previous genome wide siRNA screens have identified multiple host factors that regulate rotavirus infection (Silva-Ayala et al., 2013; Green and Pelkmans, 2016). For example, the endosomal sorting complex required for transport (ESCRT) complex has been identified as a pro-rotavirus host factor involved in rotavirus cell entry (Silva-Ayala et al., 2013). Recently, a genome-wide CRISPR-Cas9 knockout screen identified STAG2, a component of the cohesin complex, as a novel pro-rotavirus factor (Ding et al., 2018). The authors demonstrated that depletion of the STAG2 gene renders host cells resistant to viral infection through activation of the host interferon response, mediated by the cGAS/STING pathway (Ding et al., 2018). Since the CRISPR-Cas9 system is capable of disrupting targeted genes completely, the CRISPR-Cas9 screen has the potential to uncover novel host factors regulating viral infection, even for well-characterized virus (es).

### Enterovirus

Enteroviruses (EVs) belong to the family Picornaviridae and are a highly diverse group of positive-sense, single-stranded RNA viruses, including many human pathogens such as poliovirus, EV-A71, EV-D68 and rhinovirus. EVs spread through the fecal-oral or respiratory routes and cause a wide variety of diseases, including the common cold, poliomyelitis, hand, foot and mouth disease (HFMD), epidemic pleurodynia, myocarditis, herpangina, encephalitis or acute flaccid paralysis (AFP), depending on the virus type (van der Linden et al., 2015; Lugo and Krogstad, 2016).

CRISPR-Cas9 screens have contributed to identifying host factors that regulates EV infection. Diep *et al.* identified the actin histidine methyltransferase SET domain containing 3 (SETD3) as a novel host factor that controls several enteroviruses, including rhinovirus, EV-D68 and EV-A71 (Diep et al., 2019). Interestingly, SETD3 interacts with viral 2A protease regardless of its protease activity, and these interactions are required for viral replication, suggesting that the 2A protease has a previously undocumented pro-viral role unrelated to its protease activity (Diep et al., 2019). Moreover, CRISPR-Cas9 screens identified OLFML3 which support rhinovirus replication via suppression of the interferon response and multiple host proteins (e.g., MGAT5 and COG1) required for EV-D68 infection (Kim et al., 2017; Mei et al., 2021).

It is worth mentioning that the CRISPR-Cas9 tool can also be used to determine the precise role of previously identified cellular host factors for viral infection. Acyl-coenzyme A binding domain containing 3 (ACBD3) protein was originally reported to interact with EV 3A protein, which recruits PI4KB into viral replication organelles in favor of EV replication (Greninger et al., 2012; Dorobantu et al., 2014). With an siRNA knockdown approach, substantial efforts have been made to evaluate the role of ACBD3 with respect to PI4KB recruitment and EV replication, but there were discrepancies in the conclusions between the published studies. One study found that ACBD3 knockdown inhibited poliovirus replication (Greninger et al., 2012), whereas other studies observed neither inhibition of replication of EV including poliovirus, nor PI4KB recruitment (Téoulé et al., 2013; Dorobantu et al., 2014; Dorobantu et al., 2015). One possible explanation for the discrepancies among the studies is that there are incomplete and varied knockdown efficiencies of ACBD3 by siRNA. To address this, Lyoo et al., developed an ACBD3 gene knockout cell line using CRISPR-Cas9 system and found that the ACBD3 knockout inhibited replication of clinically relevant EVs, including EV-A71, poliovirus, and rhinovirus, and impaired PI4KB recruitment (Lyoo et al., 2019). This result implies that residual amounts of ACBD3 remaining after knocking down may be sufficient to support EV replication.

### Coronavirus

Coronaviruses (CoVs), which belong to the genus Coronavirus (family of Coronaviridae) are positive-sense, non-segmented RNA viruses possessing a large genome (approximately 30 kb) (Fung and Liu, 2019). A total of seven coronaviruses have been reported to be able to infect humans. Among these, four are seasonal, relatively low-pathogenicity viruses [human CoV-NL63 (HCoV-NL63), HCoV-OC43, HCoV-229E, and HCoV-HKU1], whereas the other three are highly pathogenic viruses (SARS-CoV, MERS-CoV, and SARS-CoV-2) that cause severe acute respiratory syndrome. It is generally accepted that the respiratory tract is the primary target for human CoVs to replicate, causing mild/moderate (e.g., fever and cough) to severe respiratory symptoms (e.g., lung bronchitis and pneumonia). Interestingly, infections with all seven CoVs are often accompanied by gastrointestinal symptoms, such as diarrhea and vomiting, implying gastrointestinal infection (Luo et al., 2020; Synowiec et al., 2021). ACE2, an entry receptor for HCoV-NL63, SARS-CoV, and SARS-CoV-2 is highly expressed in human intestines. In addition, recent reports demonstrated that SARS-CoV-2 was detected in the intestines of a COVID-19 patient (Xiao et al., 2020) and was able to replicate in *vitro* human intestinal culture cells and intestinal organoids (Lamers et al., 2020; Synowiec et al., 2021).

In light of the ongoing coronavirus disease 2019 (COVID-19) pandemic, intensive research efforts are currently underway worldwide to understand SARS-CoV-2 pathogenicity and discover new targets for host-directed drugs. CRISPR-Cas9 technology has been widely used to determine viral-host interactions. Indeed, to our knowledge, research outcomes utilizing genome-wide CRISPR-Cas9 knockout screens have already been published by six independent groups since the emergence of COVID-19 (Baggen et al., 2021; Daniloski et al., 2021; Schneider et al., 2021; Wang et al., 2021; Wei et al., 2021; Zhu et al., 2021). As expected, ACE2, an entry receptor for SARS-CoV-2, has been included as a hit in most published screening studies. Through these screens, many host genes related to SARS-CoV-2 infection have been identified. Interestingly, some of these genes are pro-viral factors involved in viral entry, affecting ACE2 expression or localization. Loss of HMGB1 reduces ACE expression possibly due to epigenetic repression of the ACE2 locus (Wei et al., 2021), whereas depletion of RAB7A results in reduced cell surface expression of ACE2 (Daniloski et al., 2021).

In addition, Goujon et al., carried out dual genome-wide CRISPR screens (knockout and activation) using the human lung epithelial cell line Calu-3 and identified host factors regulating SARS-CoV-2 infection (Goujon et al., 2021). It should be noted that ACE2 was the only gene that scored in both knockout and activation screens and multiple host genes that did not overlap between them were identified, likely because of different modalities in narrowing down the genes with phenotypic changes. These results strongly suggest that performing screening in both directions is beneficial for unveiling the full spectrum of host factors involved in viral infection.

## Discussion

The development of the CRISPR-Cas9 system has revolutionized medical research. In terms of enteric virus research, the CRISPR-Cas9 system has contributed to expanding our understanding of the molecular mechanisms underlying viral infections. In particular, CRISPR-Cas9 knockout/activation screens have led to discovering numerous functional needles in a mammalian genome haystack, including CD300lf and CD300ld (entry receptors for MNV), STAG2 (pro-rotavirus factor), SETD3 (pro-EV factor interacting with viral 2A protease), and HMGB1 and RAB7A (pro-SASRS-CoV-2 factors affecting ACE2 expression or localization).

Given the accuracy of CRISPR-Cas9 resulting in complete targeted gene knockout, as compared to RNAi with incomplete knockdown, reassessment of RNAi experiments using CRISPR-Cas9 gene knockout may be warranted. This is especially true for gene(s) whose phenotypes have been argued owing to discrepancies between published studies. By contrast, this feature of the CRISPR-Cas9 system is unfavorable when investigating certain gene(s) whose functions are indispensable for cell growth and/or survival, because it is difficult to establish cells lacking such genes. In this case, gene knockdown techniques such as RNAi (siRNA or shRNA) and CRISPR interference (CRISPRi) system, which we mention below, are useful for studying the effect(s) of such essential genes on viral infection.

In addition to CRISPR knockout and activation methods, which we introduce in this review, various other application tools are currently available (Brezgin et al., 2019). For instance, dCas9 fused to transcriptional repressors, such as the Krüppel-associated box (KRAB) domain, can be used in a CRISPRi assay to silence gene expression. dCas9 can be also used for epigenetic studies by fusing it to an epigenetic regulator, such as DNA methyltransferase DNMT3A. The CRISPR-Cas9 system is now an indispensable molecular tool, and we expect that this novel technology will be further employed worldwide to advance enteric virus research.
